# Effects of Health Qigong Exercises on Relieving Symptoms of Parkinson's Disease

**DOI:** 10.1155/2016/5935782

**Published:** 2016-11-07

**Authors:** Xiao Lei Liu, Shihui Chen, Yongtai Wang

**Affiliations:** ^1^Department of Traditional Sports, Beijing Sport University, Beijing, China; ^2^Department of Kinesiology, Texas A&M University-Texarkana, Texarkana, TX, USA; ^3^College of Nursing and Health Science, University of Texas at Tyler, Tyler, TX, USA

## Abstract

The purpose of this study was to investigate the effects of Health Qigong on the treatment and releasing symptoms of Parkinson's disease (PD). Fifty-four moderate PD patients (*N* = 54) were randomly divided into experimental and control groups. Twenty-eight PD patients were placed in the experimental group in which the prescribed medication plus Health Qigong exercise will be used as intervention. The other 26 PD patients as the control group were treated only with regular medication. Ten-week intervention had been conducted for the study, and participants completed the scheduled exercises 5 times per week for 60 minutes each time (10 minutes for warm-up, 40 minutes for the exercise, and 10 minutes for cooldown). Data which included the muscle hardness, one-legged blind balance, physical coordination, and stability was collected before, during, and after the intervention. Comparisons were made between the experimental and control groups through the Repeated Measures ANOVA. The results showed that PD patients demonstrate a significant improvement in muscle hardness, the timed “up and go,” balance, and hand-eye coordination (the turn-over-jars test). There were no significant differences between the two groups in gender, age, and course of differences (*P* < 0.05). The study concluded that Health Qigong exercises could reduce the symptoms of Parkinson's disease and improve the body functions of PD patients in both the mild and moderate stages. It can be added as an effective treatment of rehabilitation therapy for PD.

## 1. Introduction

Parkinson's disease (PD) is a progressive chronic disorder of the central nervous system characterized by impaired muscular coordination and tremors, and it is an age-related disease that afflicts a large number of patients globally. Individuals with PD typically have movement impairment, such as resting tremors, bradykinesia, and rigid muscles, resulting from the lack of dopamine in the extrapyramidal system [1, 2]. According to the recent statistics on Parkinson's disease, there are approximately 6.3 million people with PD in the world and more than 1.7 million people with PD in China, affecting approximately 1.7% of the Chinese population aged over 65 years (European Parkinson's Disease Association). Parkinson's disease is a painful chronical disease because it limits muscle capacity, sense of balance, motor skills, language, sleep, and daily living abilities. It may also cause psychological and sociological problems such as depression and fatigue, reducing the quality of life, and lowering self-esteem [3, 4].

People with PD need extensive rehabilitation because current medication can do little for motor function deterioration. It is crucial for PD patients to do physical therapy because they need a medical treatment and a positive influence on both body and mind. Over the past few years, achievements had been made in the therapy formulations of PD including drug therapy, gene therapy, and operative treatment. Treatment options now include rehabilitation, symptomatic treatment, protective treatment, deep-brain stimulation, and reconstruction treatment. Despite the fact that movement disorders are a main symptom of Parkinsonism, few studies use exercise therapy as an integrative approach for the prevention and treatment of PD. According to recent research studies, physical exercise helps improve clinical symptoms of PD such as postural instability, rigidity, muscle tremors, and the slowness of movement, as well as physical abilities such as muscle functions and the sense of balance [5], and more physical activity programs have been integrated into therapy treatments of PD [6, 7].

According to the statistics, falling is one of the most common and serious problems for Parkinson's patients. 66% of PD patients fall once a year and 46% encounter repeating falls [8]. This is mainly caused by symptoms such as freezes, muscle weakness, and balance problems [9, 10]. Scholars had made joint efforts to explore new methods for the treatment of PD. It had been found that physical treatments as a complement to medications could achieve superior results [11]. These physical exercises aimed to develop balance, gait, mobility, muscle control, and the capacity for independent living. Some items had been tested before and after the intervention, including akinetic symptoms, posture capacity, balance, pain evaluations, gait, rigidity, and a posture analysis. The physical treatments were mainly meant for the improvement of the patients' motor capacity, daily living ability, and muscle control [7].

Different types of physical exercise had been designed for Parkinson's patients aiming to improve patient mobility [12], balance [13], muscle control and power [14], aerobic capacity [15], and gait [16]. For instance, aerobic exercises had been widely applied to increase a PD patient's cardiac respiratory function and muscle capacity. Aerobic gait and step exercises such as walking and using a treadmill helped renew the daily life capacity, because they could develop behavioral functions and improved life quality [17]. In addition, when combining aerobic exercise with drug therapy, the effect was improved. Muhlack et al. [18] found that the efficiency of levodopa was improved after practicing aerobic exercise. Other studies had found that aerobic exercises could also protect the patients' neuro- and neural pathways [19, 20]. Aerobic exercise was found to provide patients with improved movement capacity and physical functions and ease PD symptoms [21]. Both intensive and adaptive exercise programs had been shown to improve balance and mobility in patients with PD [22].

PD patients' muscle strength is lower than that in normal people; this might be because of a loss in central stimulation [23]. According to some research, physical capacity was concerned with lower leg strength and could be improved by regular exercise [24]. For individuals with PD, immobility might also cause problems in bone density, and doing more muscle exercises could relieve this situation [25]. Lima et al. [26] suggested that mild- and medium-level PD patients undergo progressive resistance training during rehabilitation, which could increase their walking capacity and reduce the risk of falls. Hunot and Hirsch's studies [13] also found that performing strength and balance exercises could reduce the risk of falls, and patient strength and balance could be obviously increased. Oguh et al. [21] found in their research on 4,866 Parkinson's patients that regular exercise provided superior mobility and physical functioning, slowed the advancement of the disease, and reduced cognitive loss as well as nursing costs. Hove et al.'s studies [27] also showed that rhythmic exercise could significantly benefit the movement and coordination of PD patients.

In 2008, Lan et al. [28] claimed that muscle-strengthening exercises could raise patients' muscle power but that the effect on balance, gait, and movement capacity was limited. As such, the potential effectiveness of traditional mind and body harmony exercises, Tai Chi, Health Qigong, and Yoga, on treating PD was studied by some of researchers [29, 30]. The characteristics of Tai Chi and Health Qigong are slow, coherent, and aerobic low-intensity exercises that can relax body and mind. Tai Chi and Qigong are treated as a style of Chinese martial arts incorporating meditation, breathing, and physical movement. Exercising Tai Chi and Qigong serves many functions: relaxing body and mind, inducing pleasure and satiety, recharging metabolism, improving heart functions and slowing heart rates, and reducing blood pressure. Used as physiotherapy, they have always been an essential part of Chinese traditional herbal medicine [31]. Some scholars think that Qigong emerged from methods used by the ancient Chinese and, actually, it was developed on the basis of traditional Chinese medicine by controlling the movement of Qi through meridian system in the body [32]. As people in the early stages of PD can still dance, run, and walk smoothly and can also do complex movements for several minutes when they are given appropriate emotional or visual cues, Health Qigong appears to be an appropriate exercise for PD patients to improve their symptoms.

Among different types of balance exercises, Tai Chi is one of the most effective interventions that can be used by recreational therapists to improve balance and postural stability in older adults with PD [29]. Morris [33] showed that Tai Chi could improve balance, kinesthetic sense, and strength; hence, it may be prescribed as a sensorimotor agility program for patients with PD. Because Qigong and Tai Chi share many similar characteristics, the effects of Qigong on PD may resemble those functions of Tai Chi. Tai Chi and Qigong are exercises that both require the unification of the body and soul as their philosophical basis. Both are the two most popular Chinese medical exercises in improving balance, flexibility, relaxation, and postural stability worldwide [34]. Tai Chi and Qigong had been shown to improve health-related quality of life indicators and psychological health [35]. This type of exercise can relieve anxiety and depression and contribute to a sense of calm. There is growing evidence that physical and mental therapy helps eliminate negative emotions, reduce the symptoms of depression, and promote mental health. The use of mind-body therapies, such as Tai Chi, Yoga, Health Qigong, and meditation, is frequently reported as a means of coping with anxiety and depression [36].

In summary, Health Qigong is a traditional Chinese exercise that builds the practitioners mind and body by controlling the flow of Qi, comforting the body, and releasing pressure through movement. To PD patients, exercising Health Qigong is superior to taking medication because of its fewer side effects and a longer honeymoon effect. Although different traditional exercises had been attempted as treatments for the PD patients, few people use them as an alternative approach for the treatment of PD disease which the present study is trying to focus on. As one of the Chinese traditional mind and body exercises which share similar functions with Tai Chi, Health Qigong as an alternative therapy exercise integrated into regular medical treatment of PD is receiving greater attention. In order to analyze the effectiveness of Chinese Health Qigong exercises on relieving symptoms of Parkinson's disease, the purposes of this study were to conduct an experiment to investigate the effects of Health Qigong on the treatment and releasing symptoms of Parkinson's disease. The main aims of this study were (1) to design a Health Qigong exercise program for mild-to-moderate stages of PD, (2) to examine the effects of 10 weeks of Health Qigong exercise on PD, (3) to investigate the effects of Health Qigong on function of shaking, muscle hardness and elasticity, balance, and activity of daily living on PD, and (4) to specially design/modify a Health Qigong exercise form for PD.

## 2. Materials and Methods

### 2.1. Participants

Fifty-four patients diagnosed with PD, from YT Mountain Hospital, were selected as participants for this study. The inclusion criteria for the recruitment of participants were as follows: (1) mild or moderate PD, (2) ability to walk independently, (3) normal state of mental health, (4) ability to follow instructions, (5) absence of other complications, and (6) ability to participate in physical exercise. The exclusion criteria included any previous practical experience with Health Qigong, a recent or planned change in medication, and signs of a central nervous system disease other than PD, such as aphasia or dementia (as defined by the mini mental status examination). Selected participants were randomly divided into two groups. The Health Qigong experiment group included 28 patients (11 men and 17 women and average age of 65.84 ± 5.45), and the control group included 26 patients (14 men and 12 women with average age of 62.5 ± 3.13). The two groups showed no statistically significant differences in gender, age, and course of differences (*P* > 0.05).

### 2.2. Instruments

Four instruments were used to measure the outcomes of the 10-week Health Qigong program. They included the muscle hardness, the physical stability (9-holed instrument) test, and the physical coordination test (TUG and turn-over-jars tests). This study is mainly concerned with the effect of Health Qigong on PD treatment; thus, the items such as muscle hardness and elasticity, balance capacity, and physical coordination (the most common and main symptoms of PD) all needed to be measured and analyzed. The following instruments were used for collecting data: myometry (Myoton-3) for muscle hardness and elasticity and the BDW-85-II to test physical stability. In addition, balance and physical coordination were measured through (a) TUG test, (b) eye-hand coordination* (turn-over-jars)* test, and (c) one-legged blind balance test. All data was collected by the lab technicians and the project assistants.

### 2.3. Experimental Design

The participants were randomly divided into two groups. The control group received only drug therapy and participated in regular daily activities, and the experimental group participated in the Health Qigong program in addition to the drug therapy. Three measurements were conducted for the entire experiment period: pretest was conducted one week before starting the intervention of the Health Qigong exercise program, interim test was conducted four to five weeks after the intervention started, and the posttest was conducted immediately after the intervention. All instruments were reliable and were operated and recorded by the experienced technicians.

### 2.4. Intervention and Procedures

#### 2.4.1. Intervention Procedures

Ten Health Qigong movements targeted for Parkinsonism symptoms and movement patterns were selected and compiled as the Health Qigong program by the principle researcher of this study who has more than 12 years of teaching and research experiences in Qigong. She selected 10 Qigong movements from a Health Qigong program based on the nature of these movements to match the characteristics of PD condition. The 10 movements' program then was sent to five well-renowned Qigong experts in the nation for consultation. The movement selection and compilation were examined and evaluated by the distinguished Health Qigong experts, who analyzed the feasibility and potential effectiveness of the 10 movements in relieving the symptoms of PD, and were confirmed by the five experts with minor changes. The 10 Health Qigong movements are holding the hands high with palms up to regulate the internal organs (Shuang Shou Tuo Tian Li San Jiao), thrusting the fists and making the eyes glare to enhance strength (Cuan Quan Nu Mu Zeng Qi Li), and looking backwards to prevent sickness and strain (Wu Lao Qi Shang Wang Hou Qiao) from Baduanjin exercise; the “XU” and “XI” exercises from Liuzijue exercise; the bird exercise (Niao Xi) from Wuqinxi exercise; the showing talons and spreading wings (Chu Zhao Liang Chi Shi) from Yijinjing; the beginning of Heaven's creation (Qian Yuan Qi Yun) and the white crane flies high in the clouds (Yun Duan Bai He) from 12-step daoyin health preservation exercise; the dragon flying (Long Deng) from Mawangdui Daoyinshu exercise.

Each movement was to be practiced three times, taking approximately 14-15 minutes for the entire form, to improve PD's physical coordination, stability, balance, and muscle control. The Health Qigong exercise program was conducted for 10 weeks, 5 days per week, with each session lasting for 60 minutes. Each session included 10 minutes of warm-up, 40 minutes of Health Qigong practice, and 10 minutes of relaxation at the end.

### 2.5. Statistics and Data Analysis

Because PD patients were a special group, it became difficult to control the number of participants from both control and experiment groups. For different reasons, we lost some participants in the muscle hardness, action control capacity, one-legged blind balance, TUG, and hand-eye coordination (turn-over-jars) tests. According to the experiment design, the same test items between the control and experimental groups were tested during the pretest, interim test, and posttest, and the Repeated Measures ANOVA was used for the statistical analysis.

## 3. Results

### 3.1. Effect of 10 Weeks of Health Qigong on Muscle Hardness

The muscle hardness was measured on the left and right pronator teres during the pretest, interim test, and posttest, and the results were shown in Tables 1 and 2.

**Figure 1:**
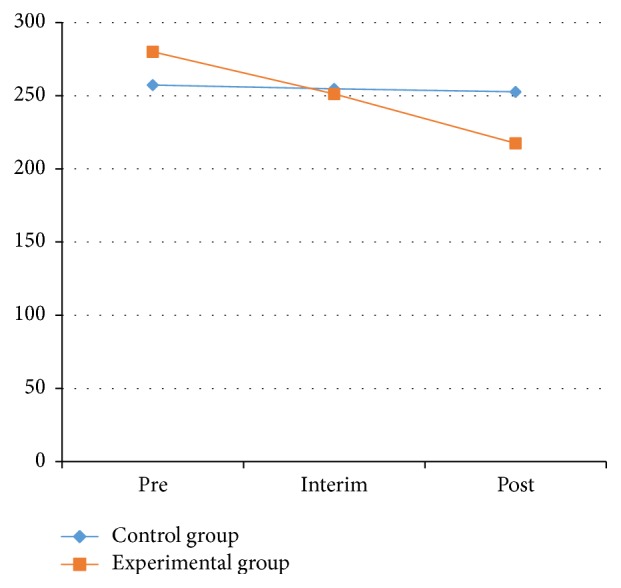
The value of left muscle hardness between control and experimental groups.

**Figure 2:**
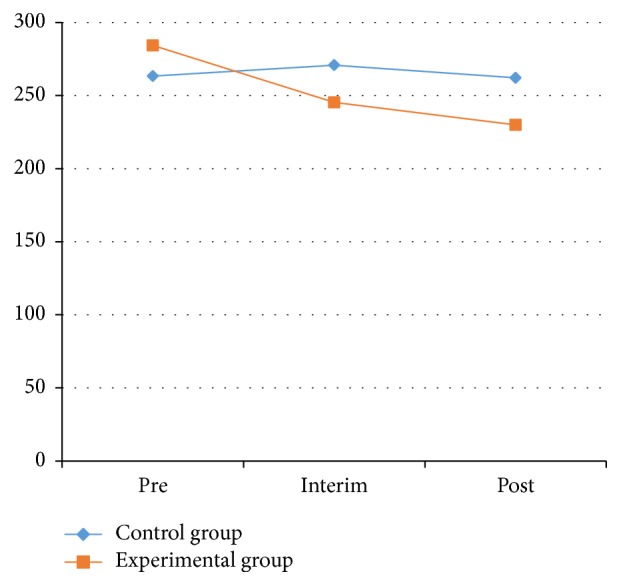
The value of left hand-eye coordination test between control and experimental groups.

As shown in Table 1 and Figures 1 and 2, the muscle hardness index of the left pronator teres decreased significantly. Compared with the muscle hardness index of the left pronator teres measured in the 2 groups at pretest, the interim test decreased from 280.00 ± 55.30 to 251.17 ± 38.29 N/m. The range declined from 284.35 ± 61.33 to 245.39 ± 40.72 N/m for the right side, with a significant level at *P* < 0.01. The range declined from 280.00 ± 55.30 to 217.48 ± 26.35 N/m from the pretest to the posttest in the left side; for the right side, the index dropped from 284.35 ± 61.33 to 229.96 ± 35.73 N/m, with a significant difference at the level *P* < 0.01.

Figures 1 and 2 indicated that after 10 weeks of Health Qigong intervention, the muscle hardness levels of the left and right pronator teres were decreased, and the muscle hardness levels declined significantly with the extension of the intervention. Health Qigong exercise can improve the muscle hardness of the pronator teres in PD patients. The data provides the evidence that Health Qigong relaxes the body and relieves stiff muscles in PD patients.

### 3.2. Effects of Health Qigong on Physical Coordination, Stability, and Balance

#### 3.2.1. Evaluating Physical Coordination in PD Patients

**Figure 3:**
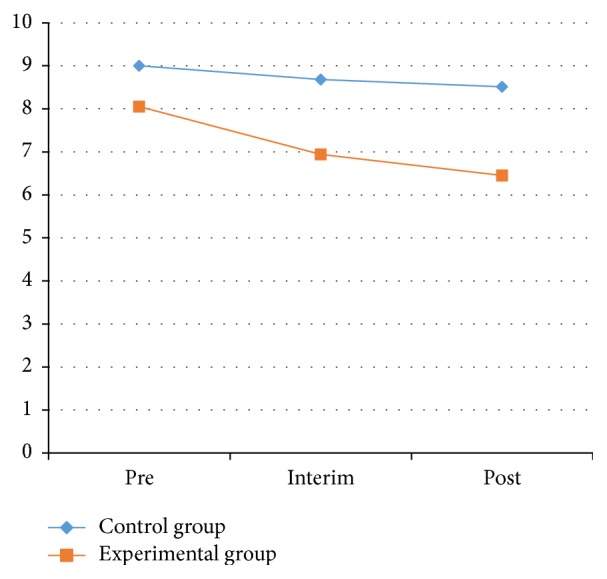
The value of left hand-eye coordination test between control and experimental groups.

**Figure 4:**
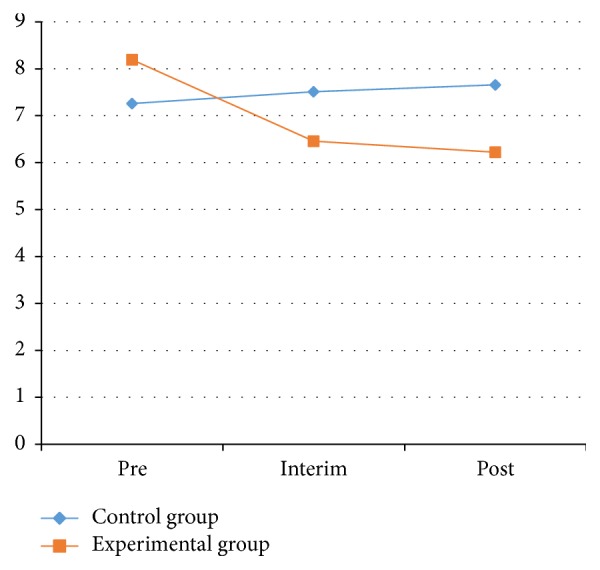
The value of right hand-eye coordination test between control and experimental groups.

*(1) Hand-Eye Coordination (Turn-over-Jars) Test*. To test the participants' upper-body control, we placed five jars (250 mL in size) in a line on a table. Each patient had to flip the jars, one at a time, as fast as they could. The test was timed with a stopwatch. The patients observed how to perform the action and complete the specifications when the time was recorded. The hand-eye coordination test results for the experimental and the control groups from the pretest, interim test, and posttest are shown in Table 2 and Figures 3 and 4.

As shown in Table 2 and Figures 3 and 4, the right-side and left-side results for the hand-eye coordination test on the control group showed no significant effect between the pretest and posttest (*P* > 0.05). The index of the left side for both groups was also not statistically significant difference (*P* > 0.05). However, the index for both groups on the right side showed significant effects between the pretest and posttest (*P* < 0.05). In the experimental group, the index decrease from the pretest to the posttest for both the left and right sides was significant (*P* < 0.05). On the left side for the experimental group, the average declined from 8.05 ± 2.90 to 6.45 ± 3.46 s; on the right side, the average dropped from 8.19 ± 4.34 to 6.22 ± 2.35 s.

The index for the experimental group before and after the hand-eye coordination test on the left side showed significant effects. Significant differences were also shown for the right side of the 2 groups between the pretest and interim test and for the left side of the experimental group between the pretest and posttest. These results illustrate that Health Qigong exercise could significantly improve the hand-eye coordination of PD patients.

**Figure 5:**
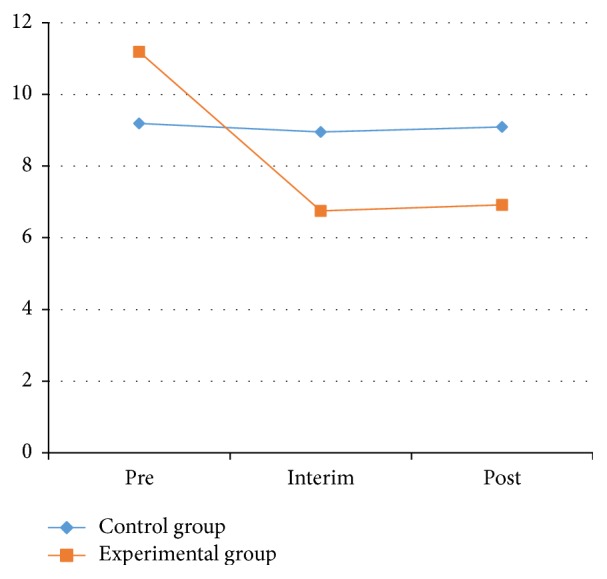
The results of right timed “up and go” test between the control and experimental groups.

*(2) TUG Test*. A timed TUG test was used to test patient's balance, gait, and stride while walking. A chair was placed against a wall, and a distance of 3 m from the chair was marked. The patient sits on the chair, and the timer is started once the patient stands up. The patient must walk to the 3 m mark, then turn around, walk back to the chair, and sit down again. The timer is stopped at this point. In the experiment, the testers recorded the completed time and observed the patient's balance, gait, and stride while walking. The results of the TUG test for both the experimental and control groups were shown in Tables 3 and 4 and Figure 5.

As shown in Table 3, the results of TUG test for the 2 groups between the pretest and the interim test and between the pretest and posttest were statistically significant (*P* < 0.01). The average TUG test index for all patients at the pretest, interim test, and posttest was 10.19 ± 0.45, 7.68 ± 0.40, and 8.00 ± 0.31 s, respectively. The pretest TUG results showed significant differences (*P* < 0.05) between the 2 groups. Significant differences were found between the interim test and posttest (*P* < 0.01) for both groups.

Table 4 and Figure 5 show that the TUG test results in the experimental group were significant between the pretest and the interim test and between the pretest and posttest (*P* < 0.01). The TUG test results for the control group showed no significant changes. In the experimental group, the average time went from 11.19 ± 2.78 s before the experiment to 6.92 ± 1.38 s after the experiment.

These data show that the stability of the experimental group had increased significantly. This illustrates that Health Qigong exercise could significantly improve physical coordination and gait in patients with PD.

#### 3.2.2. Physical Stability (9-Holed Instrument) Test to Evaluate Upper Limb Ability

The physical stability (9-holed instrument) test was used to evaluate upper limb ability; the results of the physical stability test for the experimental and control groups are shown in Table 5.

**Figure 6:**
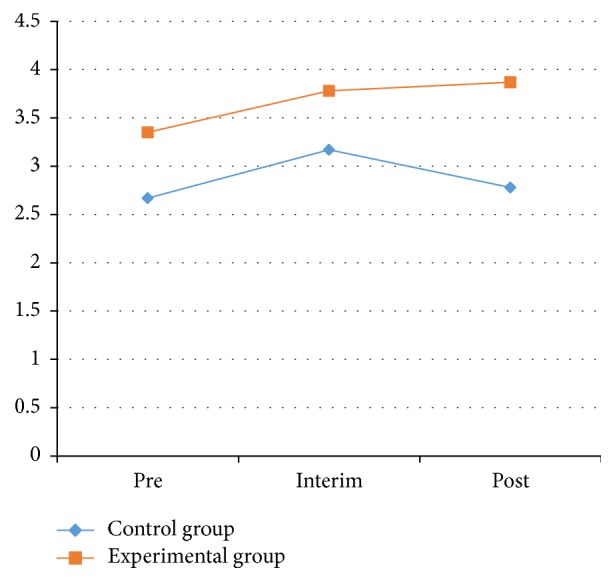
The results of left stability between control and experimental groups.

**Figure 7:**
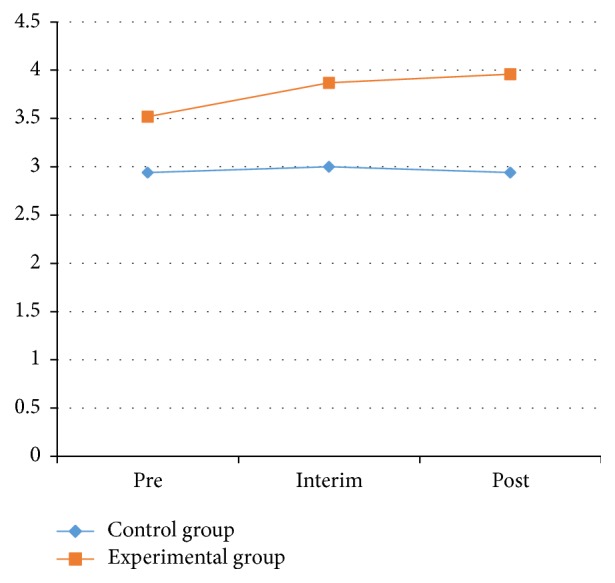
The results of right stability between control and experimental groups.

From Table 5 and Figures 6 and 7, the results for the left and right sides of the physical stability test in the experimental and control groups were not statistically significant (*P* > 0.05) between pretest and posttest. The left-side results for the control group at the pretest, interim test, and posttest were 2.67 ± 1.46, 1.86 ± 1.66, and 3.17 ± 2.78, respectively, and were not statistically significant. The right-side results for the control group at the pretest, interim test, and posttest were 2.94 ± 1.83, 3.00 ± 1.78, and 2.94 ± 1.76, respectively, also with no statistical significance.

The left-side results for the experimental group at the pretest, interim test, and posttest were 3.35 ± 1.77, 3.78 ± 2.88, and 3.87 ± 1.89, respectively, and for the right side they were 2.94 ± 1.83, 3.00 ± 1.78, and 2.94 ± 1.76, respectively. None of these results were statistically significant. These results showed that there was no significant effect on the stability of PD patients through Health Qigong exercise.

#### 3.2.3. One-Legged Blind Balance Test to Evaluate Balance

The results of the one-legged blind balance test for the experimental and control groups are shown in Table 6.

**Figure 8:**
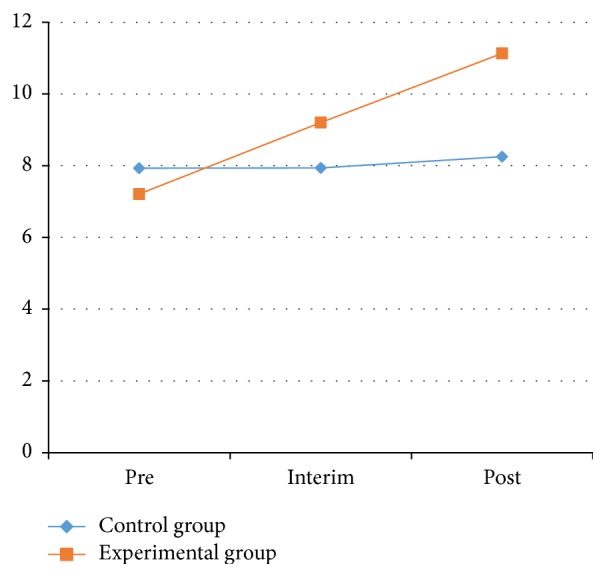
One-legged blind balance test on the left side between control and experimental groups.

**Figure 9:**
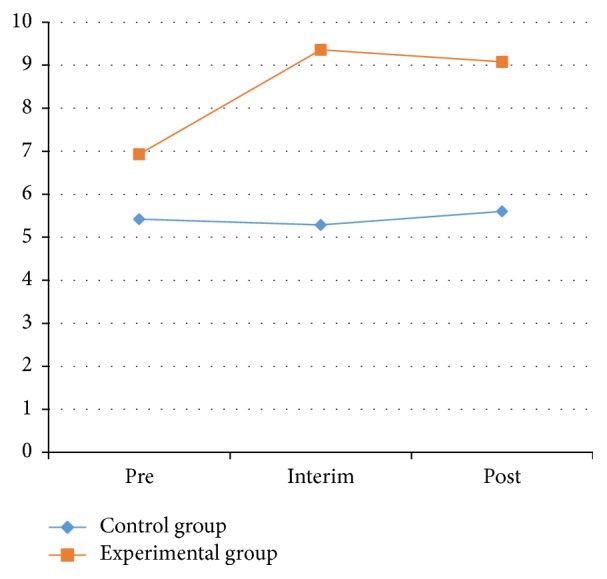
One-legged blind balance eyes test on the right side between control and experimental groups.

As Table 6 and Figures 8 and 9 showed, the left-side results of one-legged blind balance test in the experimental group showed significant differences between the pretest and posttest (*P* < 0.05). The right-side results of the experimental group showed significant changes in the process between the pretest and interim test and between the pretest and posttest (*P* < 0.05).

On the left side for the experimental group, the one-legged blind balance test average increased from 7.21 ± 4.51 to 11.13 ± 8.50 s after the experiment. On the right side for the experimental group, the average increased from 6.93 ± 3.93 s before the experiment to 9.08 ± 4.19 s after the experiment. From this data, the ability to stand on one foot had increased significantly for the experimental group. This illustrates that Health Qigong exercise could significantly improve balance in patients with PD.

## 4. Discussion

The purposes of this study were to investigate the effects of Health Qigong as a treatment on releasing symptoms of Parkinson's disease (PD). To address these purposes, four instruments or tests were used to collect data (muscle hardness, the TUG test, the turn-over-jars test, and one-legged blind balance test), and the results of measurements on muscle hardness, physical coordination, stability, and balance were examined and presented in the previous section. In the following sections, the results shown above will be further discussed item by item.

### 4.1. Muscle Hardness

An increase in muscle tension leads to a decrease in the range of motion and flexibility in PD patients. Therefore, lowering muscle tension is an essential rehabilitation goal for PD patients. Muscle tension disorder in PD patients may be a result of abnormal exercise, inhibiting cortical systems and reducing incoming sensory integration. PD patients should perform the correct relaxation exercises, rebuild the cortical system, and strengthen motor sensory system functions to lower muscle tension and hardness.

In this experiment, the differences in muscle hardness were statistically significant within various timings. The timings and groups reflected each other, and the timings were different between the experimental and control groups. The muscle hardness of the experimental group tended to decline. In other words, muscle tension in the right and left pronator teres decreased after 10 weeks of Health Qigong exercise; furthermore, the longer the time lasted, the faster the hardness level declined. Therefore, Health Qigong exercise significantly improved the muscle hardness of the pronator teres in PD patients. The longer the time is, the better the effects are.

The explanations of the significant results are as follows: the actions of Health Qigong exercise are slow and soft and the feature is relaxing across the whole process and tensional in special timing. Health Qigong practitioners tried to relax their body during the entire exercise process. In the experiment, Qigong relaxation exercises were included before each session. Patients sat on chairs with a backrest, closed their eyes, and followed the step-by-step suggestions of the coach to relax various parts of their body, eventually achieving full-body relaxation. Health Qigong exercise can increase brain electrical activity and amplitude. The status of Qigong is not between sleep and sanity, instead of a special exciting status. Qigong meditation leads the human cerebral cortex into a special status with a low psychological load and low energy consumption. This status is critical for lowering muscle tension.

### 4.2. Hand-Eye Coordination (Turn-over-Jars) Test

Hand-eye coordination refers to coordinating vision with subtle hand actions. The nerve cells of PD patients generally drop out. Because most PD patients are elderly or middle-aged, the number of peripheral nerve motor units is declared after they are 60 years old. Physiological aging of the nervous system in PD patients can affect a person's motor functions and intelligence. The aging of the movement-related cerebral cortex, subcortical structures, cerebellum, neurotransmitters, peripheral motor units, and muscles leads to poor hand-eye coordination, increased reaction times, and uncoordinated actions.

Another symptom of PD patients is slowness of movement. The initial manifestation is slowness in daily activities and movements and a prolonged reaction time. It causes slowness in subtle exercises (e.g., buttoning, using tableware, and tying laces). Slowness of movement may appear when standing up, turning over, turning, walking, and writing; walking may be clumsy, and writing may be irregular and becomes smaller when there is more to write.

In this experiment, hand-eye coordination in the experimental group tended to improve, but there were no statistically significant differences. After ten weeks of Health Qigong exercise, right- and left-side hand-eye coordination improved. The reason for the lack of statistical significance may be because the intervention time was too short. Hand-eye coordination tests the brain's ability to control the body and is an expression of neurotransmitter levels. Muscle exercise results can be seen within a short time period, but an improvement in neural functions requires more time. As can be seen from the data after 10 weeks of Health Qigong exercise, hand-eye coordination improved. A longer intervention period may significantly affect the results, because a longer time is required for neurological exercises and to repair muscles. The current results are related to the short intervention time and insignificant improvement.

### 4.3. TUG Test

Gait disorders are a typical symptom of PD. Patients generally lean forward and flex their elbows, knee joints, and lumbar spine. They walk slowly with small strides, and the walking pace is gradually reduced when the stride is increased during the walking process. An abnormal gait seriously affects the daily life of patients, so it is crucial to assess and correct a patient's gait. The TUG test has been widely used by clinicians to evaluate patients with movement disorders and elderly people's balance and mobility. Research studies have shown good reliability and validity to the TUG test in evaluating movement abilities and balance. As a quick and convenient measuring method, the TUG test is an effective method to quantitatively evaluate the functional ability of walking and has been widely applied to evaluate the balance disorders of patients. This provided the theoretical basis for the present study to use the TUG test to evaluate a patient's movement and balance ability after the Health Qigong intervention. Because the TUG test is simple and easy to conduct, it can easily be used in routine clinical examinations.

In this experiment, TUG test times decreased significantly after the 10-week Health Qigong exercise intervention. Health Qigong exercise thus significantly decreased the TUG testing time of PD patients. Furthermore, Health Qigong exercise can improve gait, stride length, and leg movement abilities. Health Qigong is a traditional Chinese exercise that forms a part of traditional Chinese culture. One of the most popular phrases used by the Chinese is, “Be earnest and down-to-earth.” Another health-related slang expression is, “Legs become old before people do”; this means that the first part of aging is in the lower limbs. Hence, Chinese people place an emphasis on the ability of the lower limbs. With aging and increasing occurrences of walking disorders, people naturally feel afraid to fall. For this reason they cannot go out and exercise. At the same time, people reduce communications and diminish contact with the outside world. This causes a vicious circle in which people age more rapidly. Health Qigong exercise can improve the functional movements ability of PD patients and reduce their risk of falls, which is particularly crucial for elderly people.

In most Health Qigong exercise, the leg movements can be seen as closed-chain exercise. Closed-chain exercise is used for maintaining and fixing a correct exercise. It improves the recognition ability of an organism and uses a reference to control movement sustainability. It is superior to chain sports training in correcting underpowered walking in PD patients. Close-chain exercise can also be used to correct missing foot heels and knee extensions. In Health Qigong exercise, when centripetal motion is characterized at the ankle, the strategies of the ankle are used to maintain the center of gravity. An effective response strategy depends on knee, hip, and trunk stability. Ankle response strategies are used to control small, slow swinging motions. The exciting sequence of muscles is from far to near. Ankle response strategies are used to maintain and restore balance in the ankle. When foot support surfaces are narrow and relatively soft, skeletal response strategies become the dominant balancing policy. The exciting sequence of muscles is from near to far. The “beginning of Heaven's creation,” bird flying, and other actions use this method to regulate balance. In addition, the actions of Health Qigong exercise such as making a fist, watching angrily, and saving the breath can strengthen core muscles to control closed-chain exercise, increase joint stress, and improve input to the trunk and lower-limb joint proprioception. All of this enhances the movement performance of patients in completing the TUG test.

### 4.4. One-Legged Blind Balance Test

Balance as a basic indicator has always been an essential aspect of physical testing, because the one-legged blind balance test is simple to operate, has a low coefficient of difficulty, is sensitive to age, and is suitable for large-scale test groups. It has been used as a balance assessment method since the last century. This method reflects the ability to balance by measuring the time taken to maintain the body's center of gravity on a single support surface when there are no visual references and relies solely on sensory organs such as the vestibular system and the coordination of muscles.

Research has shown that the aptitude of elderly people to fall is related to a deterioration of the equilibrium function, which can be delayed by physical exercise. Sports suitable for elderly people are mainly soft and slow exercises. Health Qigong, namely, Dao Yin Yang Sheng Gong in the traditional Chinese regimen, is easy to learn and can produce prominent effects. Stressing the integration of the mind, air, and body, Health Qigong is soft and slow with smooth, moderate, calm, and steady motions. When performing Health Qigong, you flex and extend, promote and demote, open and close your limbs and torso, adjust your breathing to your movements, and try to remain balanced when constantly shifting the center of gravity. As a result, the tendons and vessels throughout the body are stretched and pulled, and the main and collateral channels are dredged. Therefore, Health Qigong is an ideal sport for elderly people to improve their balance.

Health Qigong requires that practitioners maintain the gravity of the body, distinguish the true from the false, and move softly and slowly. It relies on the lumbar spinal axis to move limbs up and down, successively acting throughout each motion. Health Qigong requires no intermittent stops during the change of true or fictional actions and the transformation of gestures. It looks like flying clouds, running water, and the silkworm spinning in harmony. Senior citizens, when practicing Health Qigong, should focus more on movement accuracy in body position, arms angle, and direction and whole body coordination.

With this style, practitioners can develop the strength of the lower body muscle, stretch the thenar muscle and ligament, and improve their balance ability. Health Qigong requires symmetrical movement on the left and right sides, to stimulate the left and right brain for improved control and coordination. It is conducive to enhancing the sensitivity of the Parkinson's sense of identity awareness and the integrated processing ability of the nerve system. This is why Health Qigong exercise can have a positive influence on balance functions in patients with PD.

Health Qigong should be adjusted to the needs of each Parkinson's patient, which means that the exercise load, difficulty of movements, movement time, and frequency must be customized step by step. At the same time, Health Qigong is specially fitted with the movement of Parkinson's patients in terms of difficulty and security. It should be popularized among PD patients to increase their strength and coordination and to maintain and promote their balance ability.

In the training process of Health Qigong exercise, the body's center of gravity is constantly moving and changing directions with the upper limb movements. This effective antigravity dynamic can improve the control ability of the body to focus on the supporting surface and improve postural stability. An anticipated decrease in postural control occurs in PD patients; sometimes it may even be lost. When learning, experience and sensory input are received and muscles of the trunk and lower limbs are activated, generating expectations of stable control. The bridge reticulospinal tract in the pathway and the corticospinal reticulospinal tract are activated. First is to complete postural control and then improve postural control disorders such as propulsion. Anatomical and pathological studies of PD patients show that the disease is primarily caused by a reducing synthesis of dopamine and role of acetylcholine exciting enhancement and then there are paralysis tremors. The reduction in dopamine and the globus pallidus actively destroy the network of the central nervous system, affecting exercise cooperative movements, muscle tension, and stiffness. It is shown in agonist and antagonist muscle coordination disorder which is a major problem in starting movements, increased tremors, and muscle tension. Participating in the integration of postural control information, the basal ganglia have a close relationship with the cerebellum. They affect the automatic postural reflex, include the strategies of the hip, ankle, and stepping reflexes, and control postural control in a smooth and coordinated motion. When the basal ganglia are damaged or functional communication is disturbed, rigidity, bradykinesia, akinesia, and static or intentional tremors occur.

In this experiment, the left balance ability of the experimental group improved in comparison to the right; it first increased before returning to a downward trend, but with an overall rise. This shows that after 10 weeks of Health Qigong exercise, the ability of balance in PD patients increased, but it was not significant. This may be related to the short intervention time of only 10 weeks. The testing environment also affected the test results; for example, in the one-legged blind balance test, surrounding words and actions will directly affect balance control, so control is crucial.

## 5. Conclusion

*Areas of Improvement*. After the 10 weeks of Health Qigong exercise, the experiment compared muscle hardness on the left and right sides of the round pronator muscle, hand-eye coordination on the left and right sides, stability on the left and right sides, TUG test results on the left and right sides, and the time of one-legged blind balance test on the left and right sides. It was found that Health Qigong exercise could significantly improve PD patients' muscle hardness, functional walking capacity, hand-eye coordination, stability, and balance. However, there were no significant differences in stability. That means Health Qigong exercises can improve body functions in PD patients with early or middle stages of PD. Researchers believe 10 weeks is too short of a time period. If patients continue the exercise program for a longer time, the effects on PD will be more significant.

*Effectiveness of Qigong on PD Patients*. Qigong is a natural way to keep fit, drawing on the classical philosophy of the Yin-Yang theory and basic theories of traditional Chinese medicine regarding channels and collaterals, guidance, and breathing; the movements are perfect for patients with PD because they are simple, easy to learn, and strengthening and of low cost. Health Qigong can be promoted as a part of exercise rehabilitation therapy for PD that can reduce medical costs. It is significant to carry on their traditional Chinese mind and body exercise as a treatment for many chronic diseases. From a general survey of the experimental process, the success of the experiment is evident not only from the test data, but also from the positive feedback of the participants.

*Specially Designed Health Qigong Program Is Feasible and Suitable for PD*. The ten Health Qigong movements targeted at Parkinson's symptoms and movement patterns were selelcted and compiled as the 10-movement Health Qigong Program. The 10-movement HQ program matches the characteristics of PD condition based on the nature of these movements. The 10 movements' program was examined and evaluated by the distinguished Health Qigong experts, who analyzed the feasibility and potential effectiveness of the 10 movements in relieving the symptoms of PD, and was confirmed by the five experts as a suitable exercise program for PD patients. The results of this study have supported the effectiveness of this Health Qigong program and its feasibility as well.

*Implications for Further Study*. Many elderlies related epidemic diseases are rapidly spreading, involving a wider field and increasingly younger ages. Traditional Chinese health movements, including guided operations, Health Qigong exercise, and Tai Chi, have a long historical background with rich cultural connotations. They further have the features of being simple, easy to learn, easy to practice, secure, and obviously effective and of low cost. These features should allow for increasing numbers of people to understand, learn, and participate in their practice. Illnesses are eased while reducing the rate of sickness. Currently, the reasons people do not practice traditional Chinese movements such as Health Qigong are because it proposes high request on the professor who needs expertise and practice experience; otherwise the effect will be reduced. Although this experiment has been completed, the Health Qigong used as a treatment for PD patients is just starting. We committed to improving, mitigating, and treating Parkinson's syndrome, and we continue to strive to study traditional Chinese principles and effects, to further explore the treatment of PD, and to explore solutions of relieving its symptoms.

*The Limitations of the Study*. The experiment environment was not well controlled because of the hospital location, and there is not a separated room for the experiment, and the participants' attention and their performance might be influenced, especially the movements that need concentration. The electronic instruments sometimes went wrong and increased the testing time, which might influence participants' patience. Although we have three Qigong specialists to be responsible for the quality control (two will be the instructors and one will be supervisor during practice), more supervisors might be needed to correct participants' movement to make sure their postures are correct. The large-scale experiment with more PD patients, more scientific instruments, and better practice environment is recommended in the future.

## Figures and Tables

**Table 1:** The muscle hardness testing of the pronator teres on left and right sides during pretest, interim test, and posttest between the two groups (N/m).

	Control group (*n* = 18)			Experimental group (*n* = 23)		
	Pre	Interim	Post	Pre	Interim	Post
Left	257.28 ± 39.08	256.72 ± 38.59	252.67 ± 40.05	280.00 ± 55.30	251.17 ± 38.29^*∗∗*^	217.48 ± 26.35^*∗∗*^
Right	263.39 ± 57.23	270.78 ± 62.83	262.17 ± 61.54	284.35 ± 61.33	245.39 ± 40.72^*∗∗*^	229.96 ± 35.73^*∗∗*^

Note: *∗∗* is represented as *P* < 0.01, very significant.

**Table 2:** The hand-eye coordination test results on the right and left sides (s).

	Control group (*n* = 18)			Experimental group (*n* = 23)		
	Pre	Interim	Post	Pre	Interim	Post
Left	9.00 ± 4.90	8.68 ± 4.39	8.51 ± 3.50	8.05 ± 2.90	6.94 ± 3.38^*∗*^	6.45 ± 3.46^*∗*^
Right	7.26 ± 2.19	7.51 ± 2.24	7.66 ± 1.93	8.19 ± 4.34	6.49 ± 1.42^*∗*^	6.22 ± 2.35^*∗*^

Note: *∗* is represented as *P* < 0.05, significant.

**Table 3:** The timed “up and go” test results on the right and left sides (s).

Group	Pre	Interim	Post
Experimental	11.19 ± 0.60	6.40 ± 0.53^*∗∗*^	6.91 ± 0.41^*∗∗*^
Control	9.19 ± 0.68	8.96 ± 0.60	9.09 ± 0.46
Experimental and control	10.19 ± 0.45	7.68 ± 0.40^*∗*^	8.00 ± 0.31^*∗*^

Note: *∗* is represented as *P* < 0.05, significant; *∗∗* is represented as *P* < 0.01, very significant.

**Table 4:** The timed “up and go” test results between the control and experimental group (s).

	Control group (*n* = 18)			Experimental group (*n* = 23)		
	Pre	Interim	Post	Pre	Interim	Post
Time	9.19 ± 2.97	8.95 ± 2.81	9.09 ± 2.51	11.19 ± 2.78	6.40 ± 2.27^*∗∗*^	6.92 ± 1.38^*∗∗*^

Note: *∗∗* is represented as *P* < 0.01, very significant.

**Table 5:** The physical stability results on the right and left sides.

	Control group (*n* = 18)			Experimental group (*n* = 23)		
	Pre	Interim	Post	Pre	Interim	Post
Left	2.67 ± 1.46	3.17 ± 1.86	2.78 ± 1.66	3.35 ± 1.77	3.78 ± 2.88	3.87 ± 1.89
Right	2.94 ± 1.83	3.00 ± 1.78	2.94 ± 1.76	3.52 ± 1.83	3.87 ± 1.55	3.96 ± 2.03

**Table 6:** One-legged blind balance test on the right and left sides (s).

	Control group (*n* = 18)			Experimental group (*n* = 23)		
	Pre	Interim	Post	Pre	Interim	Post
Left	7.93 ± 4.40	7.94 ± 4.23	8.25 ± 4.27	7.21 ± 4.51	9.20 ± 5.33	11.13 ± 8.50^*∗*^
Right	5.42 ± 2.83	5.29 ± 2.99	5.60 ± 2.89	6.93 ± 3.93	9.36 ± 5.44^*∗*^	9.08 ± 4.19^*∗*^

Note: *∗* is represented as *P* < 0.05, significant.
